# The Dynamics of Ca^2+^ Ions within the Solvation Shell of Calbindin D9k

**DOI:** 10.1371/journal.pone.0014718

**Published:** 2011-02-22

**Authors:** Elad Project, Esther Nachliel, Menachem Gutman

**Affiliations:** Laser Laboratory for Fast Reactions, Biochemistry, Tel Aviv University, Tel Aviv, Israel; German Cancer Research Center, Germany

## Abstract

The encounter of a Ca^2+^ ion with a protein and its subsequent binding to specific binding sites is an intricate process that cannot be fully elucidated from experimental observations. We have applied Molecular Dynamics to study this process with atomistic details, using Calbindin D9k (CaB) as a model protein. The simulations show that in most of the time the Ca^2+^ ion spends within the Debye radius of CaB, it is being detained at the 1st and 2nd solvation shells. While being detained near the protein, the diffusion coefficient of the ion is significantly reduced. However, due to the relatively long period of detainment, the ion can scan an appreciable surface of the protein. The enhanced propagation of the ion on the surface has a functional role: significantly increasing the ability of the ion to scan the protein's surface before being dispersed to the bulk. The contribution of this mechanism to Ca^2+^ binding becomes significant at low ion concentrations, where the intervals between successive encounters with the protein are getting longer. The efficiency of the surface diffusion is affected by the distribution of charges on the protein's surface. Comparison of the Ca^2+^ binding dynamics in CaB and its E60D mutant reveals that in the wild type (WT) protein the carboxylate of E60 function as a preferred landing-site for the Ca^2+^ arriving from the bulk, followed by delivering it to the final binding site. Replacement of the glutamate by aspartate significantly reduced the ability to transfer Ca^2+^ ions from D60 to the final binding site, explaining the observed decrement in the affinity of the mutated protein to Ca^2+^.

## Introduction

Intracellular calcium plays an essential role in the transduction of most hormonal, neuronal and muscular stimuli. Cells have a multi-components calcium signaling toolkits that can be assembled to create a wide range of spatial and temporal signals. This versatility is exploited to control processes as diverse as fertilization, proliferation, development, learning and memory, contraction and secretion [1]. In general, a Ca^2+^ signal begins with the activation of a cell surface receptor or channel protein by an extracellular stimulus: virtually all tyrosine-kinase-linked receptors, G-protein-linked seven-helix transmembrane receptors, and Ca^2+^ channels trigger one or more Ca^2+^ responses [2]. The signal from the cell surface, which arrives at the intracellular Ca^2+^ store via a second messenger or via direct electrical contact, opens Ca^2+^ channels and thereby releases Ca^2+^ into the cytoplasm. The elevated Ca^2+^ concentration modulates Ca^2+^ regulatory proteins at key control points in essential physiological pathways, until the Ca^2+^ is pumped out of the cytoplasm by a Ca^2+^ ATPase.

Being of crucial importance to all living organisms, Ca^2+^ binding proteins have been the subject of many studies. One of the most prevalent Ca^2+^ binding structures is a conserved motif termed the EF-hand [3]. The EF-hand motif is widely distributed in Ca^2+^ signaling pathways and acts both in modulation, buffering and protein stabilization. The heart of the EF-hand is its Ca^2+^-binding ‘loop’, consisting of twelve consecutive residues, six of which provide direct or indirect Ca^2+^ coordination. Bordering the loop are two α-helices which serve to anchor the loop and to communicate the information of Ca^2+^ binding to distant regions of the molecule. Usually, two EF-hand sites are associated in the same protein domain to yield a highly cooperative Ca^2+^ binding system.

Various studies on EF-hand containing proteins have yielded different parameters such as specificity of the EF-hand to Ca^2+^ vs. Mg^2+^ and the rate constants of the process [2]. The association rate constants (k_on_) values of the binding process vary over a wide range for different EF-hand proteins and chelators and are considerably slower than the diffusion-controlled limit. This observation is in apparent contrast to the rapid rate of inner sphere water substitution of Ca^2+^, ranging between 10^8^ and 10^9^ sec^−1^ [4], suggesting that the binding mechanism conceals a complex sequence of events in addition to the arrival of the Ca^2+^ ion to the binding site. These events can not be elucidated by measurements of the association rate constants (k_on_), as this value is calculated indirectly from the equilibrium constants (K_eq_) and the dissociation constants (k_off_) of the process (k_on_ = k_off_ * K_eq_) [5] [6]. Thus, the derived k_on_ value incorporates the overall binding process and does not give insights on the detailed mechanism, which for Ca^2+^ ions, may be quite complex, as evident by the wide range of association rates found in the organic and inorganic world.

Several aspects of the binding mechanism of Ca^2+^ to EF-hand proteins can be derived from studies on the properties of the Ca^2+^ ion and its binding with chelators. The dehydration free energy of Ca^2+^ is as high as +362 kcal/mol [7], necessitating that any effective chelating site must replace the solvating water with strong coordinating interactions that more than offset this energetic cost. The ionic radius of Ca^2+^ is such that 7 or 8 coordinating oxygen atoms can be comfortably accommodated around the ion. Thus, these coordination numbers are most frequently observed in inorganic complexes. The kinetics of spherical metal ion binding reveals the key molecular details of the process by which a solvated ion is engulfed by chelators [8], [9]. The association kinetics are considerably faster than would be expected for a transition state involving the bare metal ion; thus the reaction mechanism is actually a stepwise replacement of solvent by ligands of the chelators, so that the ion retains approximately its normal coordination number throughout the chelating reaction.

The modeling of the complex formation should account for the flexibility of the residues in the binding site. High flexibility of the residues may slow the rate of bond formation between the ion and the protein, since the residue may have a lot of conformational space to search for the ion. On the other hand, a rigid binding site may also hinder the binding process as the ion has to perfectly align itself. What is more, it is logical to assume that the interaction of the ion with protein causes structural rearrangements in the protein. This has been witnessed in several experimental and computational studies [10], [11], [12], [13], [14], [15], [16], where removal of Ca^2+^ from the binding sites of Calmodulin (CaM) significantly altered the stability of the protein. Such conformational changes may expedite the binding process.

Beside the stepwise bond formation that seems to be the rate limiting step of the binding process, the encounter of the Ca^2+^ ion with the binding site, which is the first step in the binding process, raises interesting questions regarding the mechanism by which the ion approaches the binding site. This event may occur by a simple Brownian diffusion from the bulk. Yet it is very likely that the ion first encounters a negatively charged group on the surface of the protein and is detained in the group's Coulomb cage. In the detained state the ion may shuttle to nearby negative groups, progressing from one carboxyl to another, until it reaches the correct binding site, a concept known as the antenna effect [17]. Ca^2+^ ions, having a greater charge density and a more intricate coordination system than a proton, Na^+^ or Cl^−^ ions, poses new challenges to the suggested mechanism since the solvent-ion interactions are more complex.

Site specific mutations of Calbindin D9k (CaB) demonstrate that its surface charges play a significant role in the binding of Ca^2+^. In these experiments, charged surface residues of CaB, which do not serve as direct ligands of the Ca^2+^, were neutralized and the overall rate constant for Ca^2+^ binding (k_on_ = k_off_ * K_eq_) was shown to reduce significantly in a low ionic strength environment [5]. In physiological ionic strength, the reduction of the rate constant was much less dramatic. This observation demonstrates the crucial role of electrostatics in the binding process. Since the charges on the surface of the protein are unevenly dispersed, the detailed electrostatic potential around the protein must be considered [18]. The detailed treatment of the local electrostatic potential is of special importance at high ionic strength solution (I≥0.1 M), where the Coulomb cage radius is in the order of 2–3 water molecules. Under conditions where the electrostatic potential is screened at a short range, a negative lobe of the Coulomb cage can be broken into a set of local potential traps that may delay the ion, reducing its ability to rapidly scan the protein's surface. Conversely, one can argue that when an ion is in close proximity to the binding site, attractive surface charges not located at the binding site may deflect it from reaching the binding site, causing a decrease in the k_on_ values as compared to the same protein where these surface charges are neutralized. It is therefore clear that a more detailed evaluation of the reaction mechanism is needed.

The present research investigates the mechanism of Ca^2+^ binding to EF-hand proteins. While it is problematic to devise an experimental system suitable to monitor the dynamics of Ca^2+^ on a protein, one can use molecular dynamics to simulate the process and gain insights on the mechanism of the process. By using the simulations, the underlying processes which make up the overall binding process can be elucidated, breaking up the experimentally measured k_on_ to its constituent components. The ion detainment is a general feature reflecting the effect of local fields on the diffusion of ions. In the case of S6, where the protein does not utilize any ion for its activity, the ion detainment appears as a simple consequence of the ions interacting with the protein's electrostatic field [17]. In case of Calbindin D9k, the detainment may have a functional role.

The structure of the CaB protein [19] is of a single domain that resembles the Calmodulin's Ca^2+^ binding domain [20]. It is used as a model for many Ca^2+^ binding studies in EF-hand proteins [21] and its small size (78 residues) makes it ideal for molecular dynamics simulations. What is more, the experimental measurements on CaB mutants can be used as references for simulations on similar mutants. In the present study, we performed 100 ns long simulation of the WT protein and the E60D mutant, which is known experimentally to have a reduced affinity to Ca^2+^ [22]. The simulations show that in most of the time the Ca^2+^ ion spends within the Debye radius of CaB, it is detained at the 2^nd^ and 1^st^ solvation shells. While being detained near the protein, the diffusion coefficient of the ion is significantly reduced. However, due to the relative long period of detainment, the ion can scan an appreciable surface of the protein. This surface scanning may play a functional role, significantly increasing the surface area of the protein available for the Ca^2+^ ion, especially at low Ca^2+^ concentrations. This mechanism was demonstrated by comparison of the Ca^2+^ binding dynamics in CaB and its E60D mutant. In the WT CaB, the side chain carboxylate of E60 was witnessed to be an intermediate station for the Ca^2+^ ion arriving from the bulk, on their way to the binding residues. As witnessed from the simulations, the E60D mutant has a significantly reduced ability to transfer Ca^2+^ ions from D60 to important binding residues, thereby decreasing the overall binding affinity, explaining the experimental observations.

## Results and Discussion

### The ion binding process

The mechanism leading to the binding of an ion (or any other small molecule) to its specific binding site consists of three processes: (1) The encounter of the ion with the perimeter of the protein (usually considered as the Debye radius of the protein) (2) Wandering of the ion in the immediate proximity of the protein and (3) The final encounter with the specific binding site and the formation of the stable complex. The velocity of the first process is of a diffusion controlled reaction and varies with the concentrations of the reactants. The second process is concentration independent and the last one may reflect some rate limiting steps like preparatory conformational changes or those that follow the complex formation and stabilize its structure.

In this study we focused our attention on the 2^nd^ and 3^rd^ aspects of the binding process, using molecular dynamics simulations. The system consisted of a single protein molecule, stripped from the Ca^2+^ ion and fully relaxed, embedded in a box stretching a little more than 1.2 nm from the protein in the presence of sufficient Na^+^ and Cl^−^ ions to attain electroneutrality and maintain ionic strength of ∼0.1 M. The system contained also two Ca^2+^ ions, sufficient to saturate both binding site. The observed frequency of encounter between the Ca^2+^ ions with the protein was 1–2 per ns, in accord with the rate estimated by the Debye-Smoluchowski equation. It should be stressed that the nominal concentration of the Ca^2+^ was ∼25 µM, which is ∼3 orders of magnitude above the physiologic concentration of the ion. However, any attempt to adhere to the physiologic Ca^2+^ concentration will waste most of the simulation time waiting for primary encounters of the ion with the protein.

### The electrostatic potential surrounding the protein

**Figure 1:**
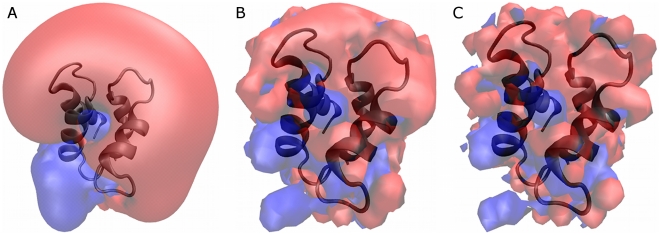
The electrostatic iso-potential surfaces of Apo-CaB. The electrostatic potential as calculated for the protein in ionic strength of 150 mM. The dielectric constant of the protein was set as ε_p_ = 2 and ε_w_ = 78. (A) 1 k_B_T surface, (B) 4 k_B_T surface and (C) 10 k_B_T surface.

The encounters of the ions with the protein are sensitive not only to the total charge of the protein but also to its precise distribution over the protein's surface [23]. The electrostatic field around CaB, accounting for the individual location of the charges and for the high ionic strength of the solution (150 mM) was calculated by the APBS program and is presented in Figure 1. Due to nature of the calculation, which is performed on a static structure and is dependent on the selected dielectric constants, some variations are expected. Thus, the results of the calculation demonstrate the trends rather than supply accurate quantitative values.

Figure 1 depicts the surface of the electrostatic potential at 1, 4 and 10 **k**_B_T levels at panels A, B and C, respectively. The 1 **k**_B_T potential (Figure 1, A) surface appears to be smooth and homogenous, located at distance of ∼10 Å from the surface of the protein (which we define as the distance to the nearest protein atom). The potential surface exhibits a major negative lobe (red) and a smaller positive one (blue). This presentation can be fairly approximated by the homogenous spherical Coulomb cage. As the Ca^2+^ ions penetrate the 1 **k**_B_T surface, the electrostatic potential increase steeply and in parallel looses its smooth appearance. At the level of 4 **k**_B_T (Figure 1, B), the surface is already bumpy and positive (repulsive) domains appear to be located below the smooth −1 **k**_B_T outer envelop, at a distance of up to 5 Å from the protein surface. Thus, as the Ca^2+^ ion is propagating towards the protein, its trajectory is affected by the local non homogenous field and specific routes are preferred. The 10k_B_T electrostatic potential (Figure 1, C) stretches to at most 3 Å from the protein surface (the first hydration shell) so while bound to the protein, the ion is trapped within fairly deep energetic wells, severely limiting its motional liberation.

### Diffusion of the ion from the bulk to the vicinity of the protein

**Figure 2:**
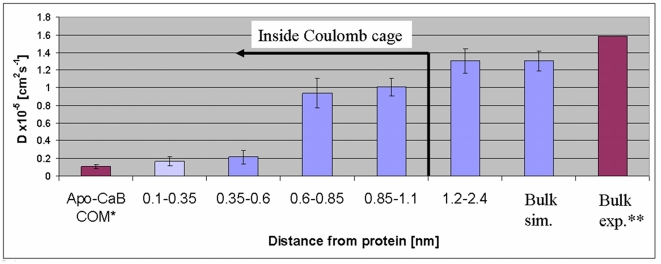
The variation of the Ca^2+^ ion's diffusion coefficient in the successive shells of Apo-CaB. The bulk columns (either from simulations or measured experimentally) refer to Ca^2+^ in protein-free solutions. * The calculated value is comparable to that of a globular protein of the same size [24], [25]. ** The calculated value is comparable with the experimental diffusion coefficient of free Ca^2+^ ions [26].

To evaluate the effect of the increasing electrostatic potential on the translational motion of the Ca^2+^ ion, we calculated the diffusion coefficient of the ion, D(Ca^2+^), as a function of its distance from the protein (defined as the distance between the Ca^2+^ ion and its nearest protein atom). The results, presented in Figure 2, demonstrate the steep variation of D(Ca^2+^) as the distance from the protein's surface changes. Ions located within the first shell (the definitions of the shells are detailed in Table 1) exhibit a diffusion coefficient of 0.166×10^−5^ cm^2^s^−1^ ±0.049×10^−5^, which only slightly exceeds the diffusion coefficient of the protein (0.110×10^−5^ cm^2^s^−1^ ±0.022×10^−5^). Ions in the second solvation shell are almost as immobile as those in the first shell. The intensity of the electrostatic potential in the second shell is in the order of 4 **k**_B_T, explaining the restriction on the translational motion of the ions at that distance from the protein. Ions located out of the 1 **k**_B_T boundary, about 10 Å from the surface of the protein, exhibit diffusion coefficient of 1.303×10^−5^ cm^2^s^−1^ ±0.140×10^−5^, that is close to the one computed for a free ion, indicating that there are no restrictions on their ability to propagate in the diffusion space.

**Table 1:** Residence time of Ca^2+^ ions near the CaB protein.

Shell distance range from the surface of the protein (nm)	τ_1_ (ps)	τ_2_ (ps)	Relative RMS error of fit
d<0.35	1.389	2670.862	0.742
0.35<d<0.6	3.611	212.193	0.663
0.6<d<0.85	1.388	22.131	0.164
0.85<d<1.1	1.072	14.341	0.057
1.1<d<1.35	0.969	13.516	0.269
1.35<d<1.6	1.145	13.774	0.183
1.6<d<1.85	1.631	15.502	0.123
1.85<d<2.1	1.876	14.695	0.223

The accuracy of the calculation is supported by the comparison of the diffusion coefficient of CaB, derived from the simulations (0.110×10^−5^ cm^2^s^−1^ ±0.022×10^−5^), with that measured for Lysozyme (0.111×10^−5^ cm^2^s^−1^) [24],[25], a protein with comparable shape and mass. Similarly, the diffusion coefficient calculated from the simulation for the Ca^2+^ in water is only slightly smaller than the experimental of 1.584×10^−5^ cm^2^s^−1^ [26], which can be attributed to the non-vanishing concentration of the ion in the simulation system [27].

### The residence time of Ca^2+^ at the vicinity of the protein

**Figure 3:**
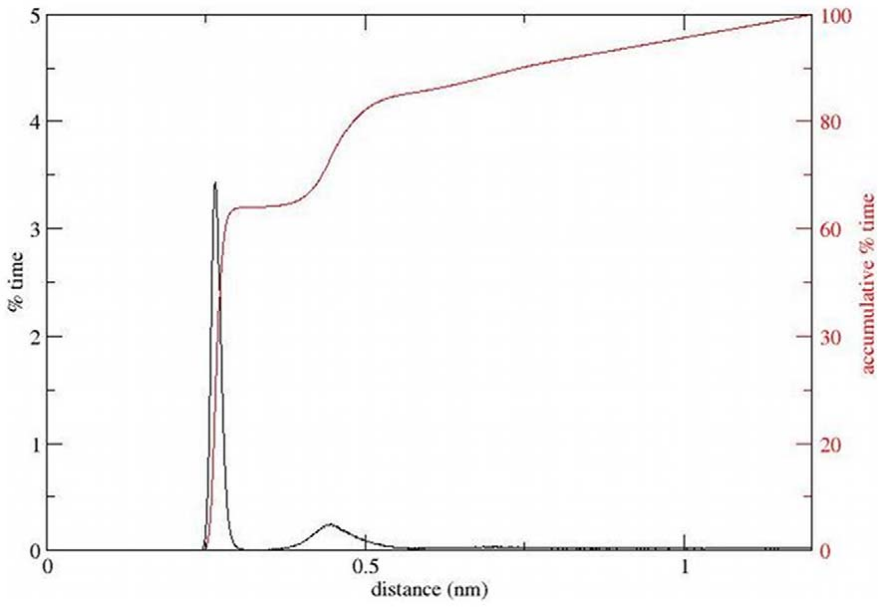
Ca^2+^ distribution around Apo-CaB. A histogram of the percentage of time a Ca^2+^ ion spends at a specific distance from the Apo-CaB, out of the total time it is within a distance of 1.2 nm from the protein (black curve). The accumulation of the histogram is presented in the red curve.

The reduction in the translational diffusivity of the Ca^2+^ ions near the protein implies that the residence time of the ions should also vary as a function of the ion's distance from the protein. During the simulation time, the Ca^2+^ ions were scanning the whole aqueous phase around the protein, but not at homogenous distribution. Figure 3 (black curve) relates the percentage of time a Ca^2+^ ion spends at a given distance from the protein, out of the total time it spends within a distance of 1.2 nm from the protein. The trace clearly indicates that the highest probability to find the ion is within the first shell. A secondary (significantly smaller) peak that corresponds with the second shell is also clearly visible. The accumulation of the calculated curve (red) indicates that at ∼65% of the time, the ion was located within the first shell of the protein, and at another 20% of the time, it was in the second shell. Close inspection of the histogram reveals hints for more remote shells, however these are hardly ordered and we have thus defined the remaining shells consecutively with a fixed width of 2.5 Å (detailed in Table 1). It should be noticed that the second peak has its maximum at ∼4.5 Å, compatible with the sum of a water molecule diameter and the ionic radius of Ca^2+^. Thus, it appears that the Ca^2+^ ion can either be directly interacting with the polar residues of CaB or through a single water molecule that bridge between them.

**Figure 4:**
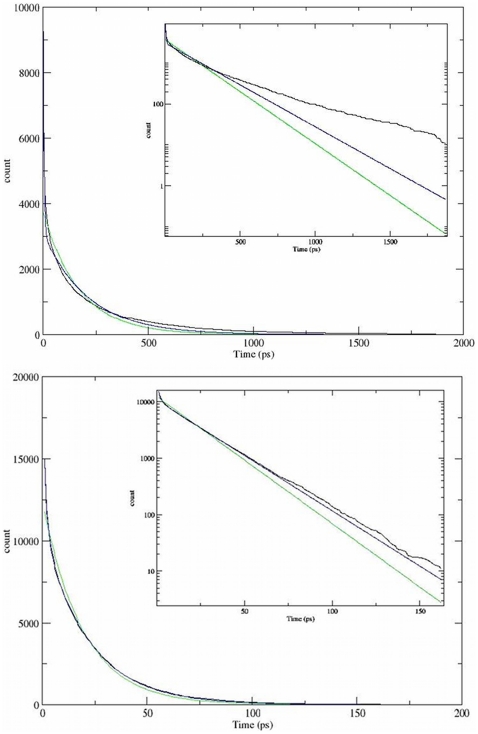
Ca^2+^ residence times. The main frame kinetics of Ca^2+^ ions residence in the 2^nd^ (top panel) and 3^rd^ (bottom panel) shell of Apo-CaB. The inset depicts the dynamics on a semi log scale. The residence time decay curves are in black. One exponential fits are in green and a bi-exponential fits are in blue.

The preferential dwelling of the ions within the first two discrete shells is reflected by the Ca^2+^ residence time. Figure 4 depicts the residence decay curves of the Ca^2+^ ions in the 2^nd^ (top panel) and 3^rd^ (bottom panel) shells. The residence decay of the Ca^2+^ near CaB, as computed from the simulations, is denoted in the black curves. As the residence time is an exponentially decaying process, an exponential fit was applied and is given in the green curve. As can be seen, in both shells the single exponential fit does not accurately describe the decay curve. This can be explained by recalling that the protein exhibits positive and negative electrostatic lobes, in which the residence time of the Ca^2+^ ion is expected to be completely different. Thus, we have performed bi-exponential fits (blue curves) and summarize the results for all shells in Table 1. Indeed, for the 3^rd^ shell and up, the bi-exponential function describes well the residence dynamics (as can be seen from the RMS errors in Table 1). Yet, for the 1^st^ and 2^nd^ solvation shells, it can be seen (according to the RMS error and visual inspection of the graphs) that a two exponential function does not accurately describe the residence curve.

All dynamics exhibit a very fast component with time constant in the order of 1–3 ps. This fast phase is attributed to Ca^2+^ molecules located in the positive lobe of the electrostatic potential. The slower phase, which relates to long residence times, is attributed to ions located within the attractive negative lobe. The ions located in the first shell exhibit an extremely long residence, in the order of 2.5 ns, while ions in the second shell dwelled in the layer with time constant of ∼200 ps. Further on along the radius vector the dwell time is reduced to ∼15 ps. On the 1^st^ and 2^nd^ shells, the residence process is more complex, due to the great inhomogeneity of the proteins surface (as can be seen in Figure 1, panels B and C). A Ca^2+^ ion in these shells is affected by the individual charges of the polarized moieties and thus the residence process becomes a complex process, which cannot be readily described by just two independent events. This local field effect is significantly reduced starting from the 3^rd^ shell and outwards. Ca^2+^ ions in these shells are affected by the charges on the protein as a whole rather than the individual moieties.

It is of interest to compare the residence time of ions leaving towards the bulk to those leaving towards the protein. The flux from the first shell is, naturally, only to the second shell. The residence time of ions in the second shell flowing inwards is 2.5 times larger than the residence time of ions flowing outwards. This reflects the high gradient of the electrostatic potential at the ∼0.3 nm range from the surface of the protein. Beyond the 2^nd^ shell, the inward and outward ion fluxes are essentially the same with a value comparable with the residence time.

### Propagation of the Ca^2+^ within the protein's Coulomb cage

#### i. The lateral translation of the ions in the respective solvation shells

**Figure 5:**
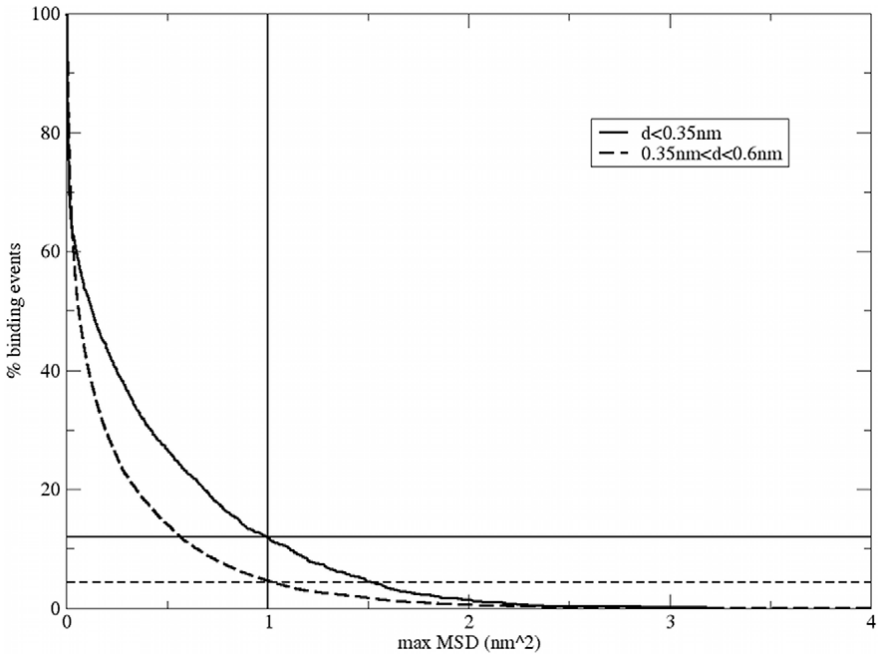
The diffusion mobility of Ca^2+^ ions on the surface of the protein. The figure relates the translational motion of Ca^+2^ ions expressed by the MSD of a Ca^2+^ ion as a function of the time the ion remained with the first (solid line) or the second (dashed line) shell of Apo-CaB. The translational motion of the ion was corrected for the rotation and translation of the protein. Straight lines indicate events where MSD > = 1 nm^2^.

The approach of Ca^2+^ ions towards the protein is accompanied by decrement of its diffusion coefficient and longer residence time. The lower diffusivity tends to reduce the translational motion of the ion while the longer residence time will allow it more time to propagate within the shell. The combined effect of the two terms on the ability of ion to scan its immediate vicinity can be evaluated by the function presented in Figure 5. The figure is a histogram of the maximum mean square deviation (MSD) of the Ca^2+^ ions while residing in the 1^st^ (solid) and 2^nd^ (dashed) solvation shells. For this calculation we compiled the data of all CaB simulations (totaling 2 µs) screening for the events where the Ca^2+^ ions resided in a specific shell. The curves in the figure represent the percentage of events (within a specific shell) in which the Ca^2+^ ion propagated (measured as MSD) more than the value in the abscissa, relative to the surface of the protein.

The results of this analysis clearly imply that the fraction of Ca^2+^ ions that propagated more than 1 nm (MSD ≥1 nm^2^) while in the 1^st^ solvation shell (∼13%) is larger than that of the ions in the 2^nd^ shell (∼4%). Considering that the length of a glutamate side chain is ∼0.4 nm, a ∼1 nm displacement cannot be achieved through a “piggy back” riding over the rotameric space of the residues. There must be a net motion on the surface of the protein. In contrast, when the ion is detained in the 2^nd^ solvation shell, it moves more than 1 nm only 4% of the time (dashed line). Thus, despite the restricted diffusion coefficient of the ion in the first solvation shell, the long dwell time allows the ion to translate over the protein's surface.

Based on these observations we conclude that ions located in the first solvation shell are mobile enough to skim over the surface of the protein and search for alternative sites. Thus the Ca^2+^ ion may end up in quite a different site than the one it first encountered.

#### ii. The encounter of Ca^2+^ ions with Apo-CaB

**Figure 6:**
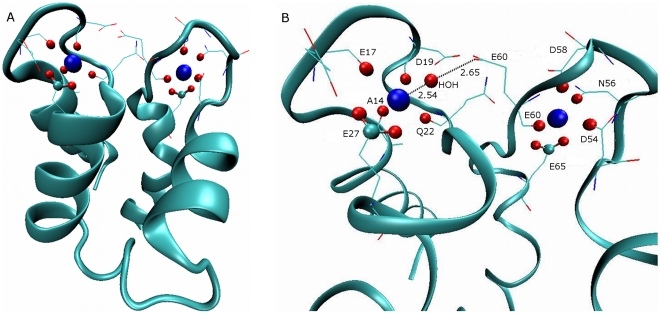
The structure of Holo Calbindin D9k. The CaB (backbone cartoon) with two bound Ca^2+^ ions (blue VDW) as taken from the crystal structure 3ICB. The binding residues are denoted as lines. Protein atoms closer than 0.3 nm are denoted as CPK (oxygens in red, carbons in cyan). Frame A depicts the whole protein. Frame B depicts the binding sites with the names of the binding residues. The E60 hydrogen bonded water oxygen that coordinate site I Ca^2+^ is also shown (in CPK) along with the relevant distances.

Figure 6, frame A, shows the overall structure of Calbindin D9k with the two bound Ca^2+^ ions, as taken from the crystallographic structure by Szebenyi and Moffat [28]. A detailed view of the binding sites, shown in frame B, demonstrates that residue E60 takes part in the coordination of the Ca^2+^ ions on both sites (either directly or via a water molecule). During the molecular dynamics simulations, the protein was ‘stripped’ from the Ca^2+^ ions and allowed to relax into a solution structure of the Apo state, both for the WT and the E60D mutation. This observation is in accord with our previous report that the Amber 94 force field indeed retains the structure of CaB intact, both in the Apo and Holo states [29]. On accumulation of 1 µs for each of the structures, the protein exercised structural fluctuations, but basically retained its shape without any deviations that should be accounted for (data not shown). Accordingly, we can discuss the motion of the Ca^2+^ ions without the need to evaluate the changes experienced by the protein.

The mechanism by which a Ca^2+^ ion propagates on the surface of the protein was investigated by examining the encounters of the Ca^2+^ ions with the individual residues on the surface of the CaB protein. The formal concentration of Ca^2+^ in the simulations was 25 mM, leading to an average frequency of one encounter every 1.3 ns. These encounters, where the ion came to contact with a residue on the surface after diffusion in the bulk, are defined as primary encounters. We define secondary encounters as events where the Ca^2+^ ion came to contact with a residue due to transfer from another surface residue, without leaving the first solvation shell. The compilations of all these events are given in Tables 2, S1, and S2.

**Table 2:** Total binding times, primary hit count and secondary hit count of Ca^2+^ ion to Apo-CaB residues, calculated from ten 100 ns WT and ten 100 ns E60D simulations.

	WT			E60D		
Residue	Binding time (ps)	Primary encounters	Secondary encounters	Binding time (ps)	Primary encounters	Secondary encounters
E4	4681	5	0	3282	4	0
E5	1	1	0	0	0	0
E11	3769	6	1	1331	1	1
Y13	0	0	0	1	0	1
**A14**	0			0		
A15	0	0	0	1080	5	1
K16	2966	4	2	3296	5	2
**E17**	139336	51	10	269478	57	8
G18	1434	5	1	2878	4	0
**D19**	15339	10	7	1520	8	2
A21	4491	4	2	45288	6	3
**Q22**	67512	52	16	176169	37	18
L23	55	19	1	0	0	0
S24	1463	3	8	21335	15	11
E26	18961	6	5	18842	8	6
**E27**	24807	4	5	29762	6	5
K41	657	0	3	722	3	1
G42	841	9	2	1935	3	1
P43	89505	16	11	31363	8	2
S44	3254	4	10	1469	2	2
T45	1874	0	2	1	0	1
D47	62462	37	11	40492	25	4
E48	176802	55	7	92731	10	7
E51	159179	62	14	130802	42	8
E52	12894	15	3	3911	9	1
L53	0	0	0	2	0	1
**D54**	96269	17	22	94526	19	4
K55	1427	7	3	8392	16	2
**A56**	36670	28	6	85218	17	11
G57	73675	28	11	7737	11	2
**D58**	191748	58	19	385846	88	12
F59	20194	9	10	486	2	7
**E60**	269285	71	35	*527753	*165	*27
S62	1033	14	16	3118	175	14
E64	31634	26	11	17635	28	2
**E65**	157857	26	12	21236	24	7
Q67	0	0	0	1	0	1
K72	0	0	0	56253	15	2
I73	22122	6	3	7630	15	2
S74	2531	16	2	11640	33	6
Q75	80	1	0	1238	2	1

Table 2 lists the compilation of the total time of contact between the ion and the residues (expressed as number of snapshots) and the number of primary and secondary encounters made with the residues. The data summed in the table corresponds with 1 µs of total simulation time (10 independent runs lasting 100 ns) for each of the WT and E60D mutant. During this period the Ca^2+^ ions were free in solution for 44% of the time and, when bound to the protein, interacted simultaneously with (average) ∼1.6 nearby residues. Inspection of Table 2 reveals that not all residues were attractive to Ca^2+^ ions by the same extent. Few residues experienced many encounters and accumulated extended contact time (for example: D58, D60, D17, E19) while others, of equal charge, hardly encountered with the ion (E4, E5, E11). Inspection of the table reveals that the high frequency of encounters, and a long binding time is not limited to the binding sites (marked by bold letters). It should be mentioned that A14, which in the crystalline state of the protein is a ligand to the Ca^2+^ in site I, did not make a single contact with the Ca^2+^ during the whole simulation.

Comparison between the abundance of primary encounters to the secondary ones reveals that the ratio between the total secondary encounters to the total primary encounters per residue is ∼0.3, meaning that of all contacts made by the ion with the protein about one quarter are mediated through a previous encounter of the ion a with some other site on the protein rather than coming directly from the bulk. The average dwell time of the ions on the binding residues were widely spread; some residues (like E65, P43) retained contact for few ns, while other residues (like S62) release the Ca^2+^ ion within few tens of ps.

Examination of the accumulative time of contact of the residues associated with binding sites I and II reveals that from all residues making binding site I, only E17, and to a lesser extant Q22, were sufficiently attractive to retain the ion for 14% and 7% of the total simulation time, respectively. The residues of binding site II appeared to be more reactive both in accumulated contact time (up to ∼29% for D58 and ∼40% for E60/D60) and in the number of primary and secondary encounters. These findings are correlated with experimental indications that site II is occupied first during the binding process [22]. Finally, there are residues that are not associated directly with the specific binding sites (P43, D47, E48, E51) that are attractive both for primary and secondary encounters, suggesting that they may function in passing the ion between nearby residues.

A further breakdown to the specific binding atoms is given in Tables S1 and S2. The first lists the encounters of Ca^2+^ with the oxygen atoms of backbone carbonyls while the latter reports encounters with the more polarized side chain oxygen atoms. The overall ratio between secondary and primary encounters for all the protein oxygens is 0.38, which is somewhat higher than 0.3 ratio observed at the per-residue resolution. This implies that intra-residue Ca^2+^ transfer occur quite frequently. The overall ratio between secondary and primary hits for carboxylates and carbonyls is 0.33 and 0.55, respectively.

The approximated rate of encounter between the Ca^2+^ ions with calbindin, derived by the Debye-Smoluchowski equation is ∼3×10^10^ M^−1^s^−1^ and the apparent rate constant at 25 mM Ca^2+^ will be ∼0.8×10^9^ s^−1^ or encounters at average intervals of 1.3 ns. The value derived from the number of primary hits that occurred during the 2 µs trajectories is quite comparable, ∼1.3 ns. Thus, the encounter of the ion with the surface of the protein is compatible with that of a diffusion controlled reaction and depends on the concentration of the Ca^2+^ ions in the solution. Once a primary encounter occurs, the following secondary encounters are independent of the free Ca^2+^ concentration and occur at average intervals of ∼4 ns. Under physiologic conditions, were the free Ca^2+^ concentration is few µM, the time interval between primary encounters will be stretched to the µs time frame. However, many of the Ca^2+^ ions that encounter the protein's surface will be transferred to a nearby site (secondary encounter) within the ns time frame. Thus, as at physiologic Ca^2+^ concentration, the primary encounters will serve as efficient donors to the nearby sites, rendering the protein's surface as a collecting antenna, and increasing the surface of the protein which is scanned by the ions.

### The Binding of Ca^2+^ to the E60D mutant of Calbindin D9k

The measured rate constant for the binding of the Ca^2+^ ion to the specific binding sites (k_on_ = 2×10^7^ M^−1^s^−1^) [30] implies that within the present simulation time, we are still far from the formation of the final stable complex as determined by the X ray diffraction of the crystal. Consequently our simulation can reflect only on the initial events associated with the binding of Ca^2+^ to the specific sites. Still these simulations are sufficient to reveal how a single mutation can alter the reaction pathway.

The functional role of the Ca^2+^ shuttling mechanism can be evaluated in the CaB itself by comparing the WT simulations with the simulations of the E60D mutant. This conservative mutation has a highly similar structure to the WT CaB, yet at physiologic ionic strength its affinity to Ca^2+^ is reduced by ∼8 fold [22]. This effect was partly attributed [22] to a more labile water molecule that binds site I Ca^2+^ (the left binding site, as seen in Figures 6). As shown in Figure 6B, the side chain of E60, as determined for the crystalline protein, indirectly coordinates the Ca^2+^ ion in site I via a water molecule. This water molecule is bound to the site I Ca^2+^ and hydrogen bonded to the side chain carboxylate of E60. The shortening of the E60 side chain by a carbon in the E60D mutant increased the length of the hydrogen bond from ∼2.65 Å to ∼2.95 Å, making the water molecule more labile. Since the experimental evidence indicates that both sites show similar reduction in affinity and that site II is occupied first (and that the binding is a cooperative process), we wished to investigate the mechanism leading to the lower affinity of site II.

The interactions between the Ca^2+^ ion with its surrounding ligands are detailed in Figure 6 frame B. The ion in site I is bound to the carboxylate of E27 and the backbone carbonyls of A14, E17, D19 and Q22. These coordinating species are in good agreement with the NMR structure 1B1G [31], with the exception that in the NMR structures the carbonyl of G18 and one of the D19 carboxylate oxygens can also serve as binding atoms. Site II Ca^2+^ is bound in the crystal structure to the carbonyl of E60, a single carboxylate oxygen of D54, a single carboxylate oxygen of D58, the amide oxygen of N56 and the carboxylate of E65. In the NMR structures, only D54 and E65, with one or two carboxylate oxygen coordination appear in all structures. D58 carboxylate and E60 carbonyl appear in the majority of the structures and N56 is missing altogether, replaced by the carbonyl of G58 or E59.

In order to resolve these apparent discrepancies, we have to consider that both crystallographic and NMR structure determination involve inherent biases. In crystallography, the crystal structure is created in non-physiological conditions. The NMR structures reflect some sort of average, and not necessarily a structure that existed in the solution. Both methods carry a bias caused by the FF used to refine them. In a recent study by Paquin et. al., NMR was used to measure the S^2^ order parameter of amide carbonyl and carboxyl groups in the side chains of aspartic acid, asparagine, glutamic acid, and glutamine of Ca^2+^-loaded calbindin D9k P43G [32]. Since the S^2^ order parameter is model independent, and measured in solution, some of the biases described above are eliminated. The results of Paquin et. al. show that the Ca^2+^ binding residues (as witnessed from crystallographic and NMR structures) are associated with high S^2^ values in the range of 0.68<S^2^<0.94. It can also be witnessed that three of the binding carboxylates, E27, D54 and E65, show exceptionally high S^2^ values of 0.836±0.041, 0.938±0.047 and 0.913±0.056, respectively, indicating an extremely low motional liberation. These values are significantly higher than those of the other binding residues, ASN56 and D58, which exhibit values of 0.685±0.037 and 0.681±0.075, respectively. For E27, there was a consensus between the crystal and NMR structures that it binds Ca^2+^ in a bi-dentate manner, using both of its carboxylate oxygens. The high S^2^ values of D54 and E65 suggests that D54 and E65 both bind the site II Ca^2+^ ion using both their carboxylate oxygens. This will form tight binding with very little motional liberation of both residues.

This conclusion also conforms well to the fact that these two residues appear as binding residues in all crystallographic and NMR structures. The lower S^2^ values of the ASN56 and D58 residues indicate that they bind the Ca^2+^ less tightly. For the ASN56 this is probably due to the weaker electrostatic attraction of the amide carbonyl oxygen. For the D58 this is probably due to being bound by only one of the carboxylate oxygens (as is also seen in the crystal structures and some of the NMR structures). To conclude, the experimental evidence suggests that the key residues involved in a stable Ca^2+^ binding in site II are D54 and E65.

As can be seen in Table 2, residue 60 (either aspartate or glutamate) is the main Ca^2+^ attractor in CaB. This is mostly due to its side chain carboxylate (as can be seen in Tables S1 and S2), even that the side chain is not involved in the final coordination of the bound site II Ca^2+^. In the case of the WT protein, the carboxylate moiety of E60 retains the Ca^2+^ for ∼25% of the total accumulative time, with an average dwell time of ∼2.4 ns.

**Figure 7:**
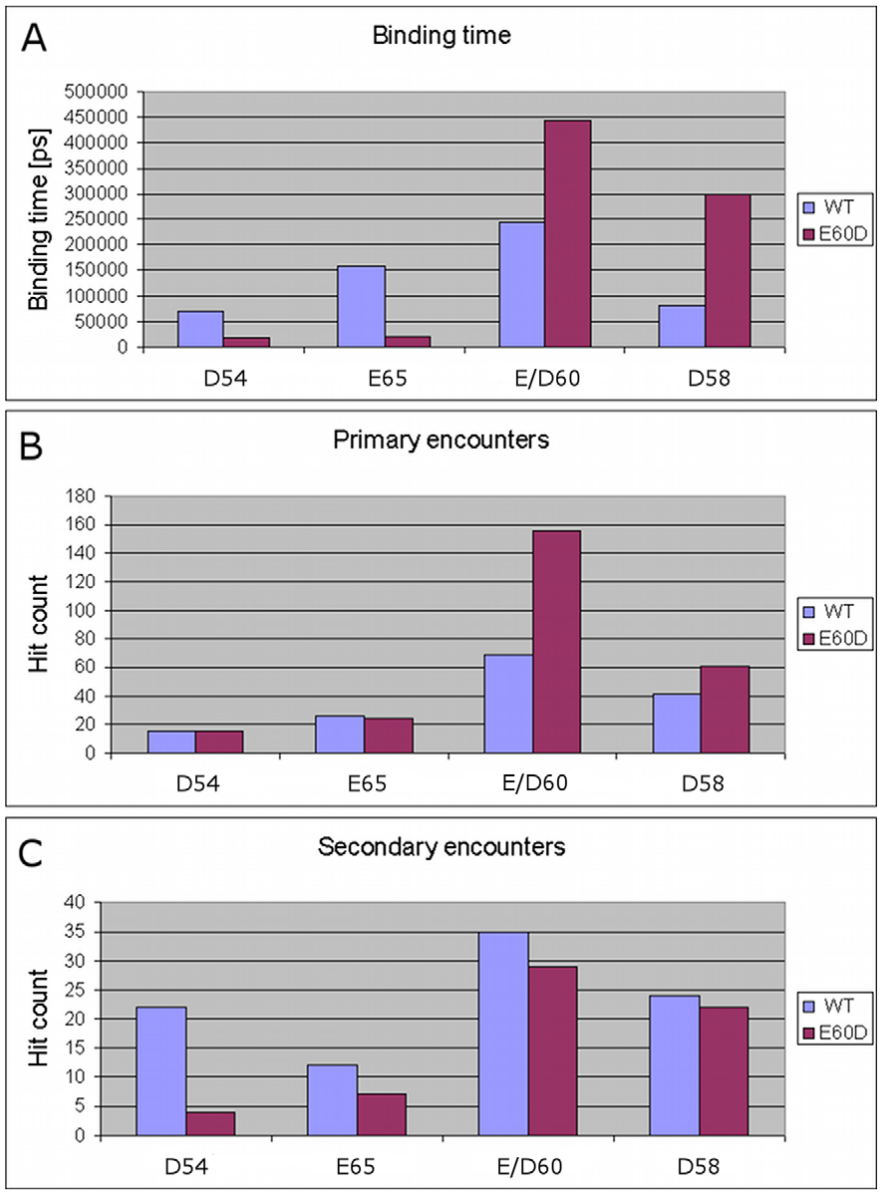
The Ca^2+^ binding characteristics of specific anionic residues Associated with the Ca^+2^ binding sites. (A) Total binding time, (B) number of primary hits and (C) number of secondary hits. Data is presented for the WT (blue) and E60D (red).

Figure 7 summarizes the total binding time (panel A), primary encounters (panel B) and secondary encounters (panel C) of the side chain carboxylates of D54, D58, E60/D60 and E65 in the WT and E60D mutant (as summarized in Table S2). As can be seen in the top panel, the replacement of E60 by aspartate enhances the binding time of the D60 carboxylate with the ion. As can be deduced from Figure 7 (middle and bottom panels), the increased binding time was due to increased primary encounters, with no change in the average dwell time (∼2.4 ns). For D58, the increase in total binding time was even more dramatic, which was also due to increase in primary hits. As can be calculated from table S2, the average dwell time increased from ∼1.3 ns to 3.6 ns.

In contrast to the increased affinity shown by D58 and D60, the two key residues for Ca^2+^ binding in site II, D54 and E65, showed decreased affinity for Ca^2+^, possibly explaining the experimentally measured reduced affinity of the E60D mutant to Ca^2+^. In the WT protein, the carboxylate side chain of D54 shows a relatively moderate overall binding time to the ion (∼7%). The E65, on the other hand, shows a relatively high binding time (∼16%). It is interesting that in the mutant both these residues show substantial decrease in the overall binding time. As demonstrated in Figure 7, panel A, for D54 there is a ∼4 fold decrease in the binding time and in the E65 there is a ∼7 fold decrease in the total binding time. A more in-depth look at the mode of encounter for these residues shows that for both D54 and E65 the main cause for the reduction in total binding time is a significant reduction in secondary hits. For E65, there was also a decrease in the dwell time.

**Figure 8:**
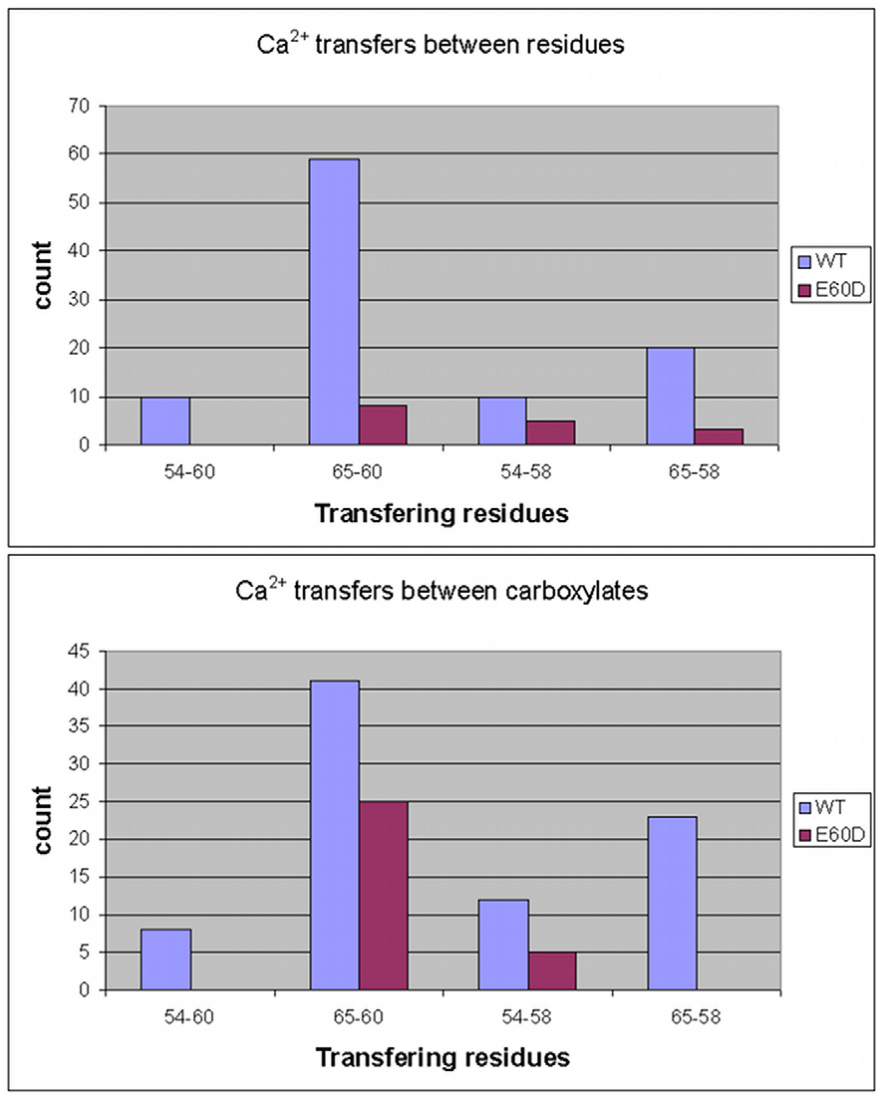
Inter-residue Ca^2+^ transfers. Number of Ca^2+^ transfers between residues 58 and 60 with residues 54 and 65. The top panel counts the Ca^2+^ transfer between the whole residues where the bottom panel counts the Ca^2+^ transfer between the carboxylates. All events, in any direction were counted for both the WT (blue) and E60D (red) simulations.

Figure 8 top panel, shows the amount of Ca^2+^ mediated interactions between specific pairs of residues: D54 and D58, D54 and D/E60, E65 and D58, E65 and D/E60. It can be seen that there is a significant reduction in Ca^2+^ transfers between these residues, which can quantitatively explain the reduction in binding time observed for D54 and E65. However, when looking at the Ca^2+^ transfers between the carboxylates of these residues, it can be seen that the Ca^2+^ transfer between the carboxylates of residues D60 and E65 was only mildly reduced. This discrepancy can be explained by the major reduction observed between the E65 carboxylate and the D60 carbonyl. It is also worth noting that a 2-fold increase in Ca^2+^ transfer is witnessed between D60 and D58 carboxylates (data not shown).

All the above data leads to a mechanism by which in the WT CaB, Ca^2+^ arrives from the bulk mainly to the carboxylate side chain of E60. E60 can than transfer the Ca^2+^ ions to the key binding residues D54 and E65. Shortening the length of its side chain significantly reduces the efficiency of these Ca^2+^ transfer reactions and alternatively increases the Ca^2+^ transfer of D60 with D58. Effectively, in the E60D mutant, both D54 and E65 become “starved” for Ca^2+^ and the overall binding affinity in site II diminishes significantly.

For D54, the reduction in Ca^2+^ binding is due to a reduction of Ca^2+^ transfers between its carboxylate and the carboxylates of D60, D58 and E65 (the latter is a consequence of its own decreased affinity).

For E65, the reduction is due to a reduction in both inter-carboxylate interactions with the carboxylates of D60 and D58, and also from a reduction in its interaction with the backbone carbonyl of D60. The latter case can be explained by the increased level of Ca^2+^ transfers observed between these three species (the carboxylates of D60 and D58 and the carbonyl of D60), reducing their availability to transfer Ca^2+^ to E65.

### Concluding remarks

The interaction of the Ca^2+^ ion with the protein starts when the ion encounters the Coulomb cage of the protein. Out of the Coulomb cage the ion is unaware of the protein, having a diffusion coefficient of a free ion. Once it is within the cage, the motion is restricted by the local electrostatic fields and its diffusion coefficient decreases as it gets closer to the protein. Although the mobility of the ion is hindered by the decreased diffusion coefficient, as the ion gets closer to the protein's surface, its residence time increases, enabling it to skim over longer distances in the 1^st^ shell than in the 2^nd^ one. Thus, the sites first encountered by the Ca^2+^ ion need not necessarily be the final binding site. The Ca^2+^ can propagate on the surface by hopping from residue to residue.

For carbonyls, which are weaker binding species, the Ca^2+^ binding mostly occurs by hopping from a nearby residue. For carboxylates, the encounter occurs mainly from the bulk, with 33% from nearby residues. In low Ca^2+^ concentration, when encounters are rare, the secondary encounters may significantly increase the protein surface available to the Ca^2+^ ion, thus making the region around the binding site a collecting antenna.

On average, the ion on the surface is interacting with more than single residue, keeping it bound for a long time, reducing the probability of getting lost to the bulk.

The Ca^2+^ shuttling mechanism was observed in simulations of Calbindin D9k where the main Ca^2+^ attractor is the side chain carboxylate of E60, which is not a part of the binding site, according to experimental structures. Thus, this site only serves as a midway station for Ca^2+^ arriving from the bulk on their way to the proper binding site. In the calbindin E60D mutant, the ability of D60 to transfer the Ca^2+^ ion to key binding residues in site II (D54 and E65) is significantly reduced, which we suggest as a possible cause for the reduced affinity observed in experiments.

## Methods

The number of simulations that were carried out, their lengths and compositions are given in Table 3.

**Table 3:** Simulations' details and composition.

Name	Duration of simulation (ns)	Number of simulations	Number of water molecules	Ionic composition
Ca^2+^ in water	2	20	2174	1Ca^2+^, 2Cl^−^
WT Apo-CaB	100	10	4472	2Ca^2+^, 8Cl^−^, 11Na^+^
E60D Apo-CaB	100	10	4317	2Ca^2+^, 7Cl^−^, 10Na^+^

The force field selected for the simulation was Amber94 [33] ported by Sorin and Pande [34]. To optimize the interaction of the Ca^2+^ ion with carboxylate we modulated the Lennard–Jones parameters as described in [35], using the values: σ = 0.300616 nm; ε = 1.87 KJ·mol^−1^. The TIP3P [36] water model was used in all simulations. The simulations were performed using the GROMACS 4 package of programs [37], [38], [39], [40]. The WT Calbindin protein structure (PDB code 3ICB) given in the Protein Data Bank(PDB) [41] was determined by X-ray crystallography at 0.23 nm [28]. In order to simulate a Ca^2+^ depleted CaB (Apo-CaB), the two Ca^2+^ ions were removed from their binding sites and added at random locations. The water box extended to at least 1.2 nm from the molecule, for CaB simulations, and at least 2.0 nm from the Ca^2+^ ion for the free Ca^2+^ simulations. D, E and LYS residues were charged to reflect their state at physiological pH. Electroneutrality and physiological ionic strength of ∼0.150 M for the CaB simulations and 0.1 M for the free Ca^2+^ simulations were reached by adding appropriate amounts of Na^+^ and Cl^−^ ions to the simulation boxes (as detailed in Table 3). Prior to the dynamics simulation, internal constraints were relaxed by energy minimization. Following the minimization, an MD equilibration run was performed for 20 ps under position restraints. Finally, before all the production runs, further unconstrained equilibrations were performed for 200 ps. After the equilibration, an MD production run was performed for extended time frame, as described in Table 3. The time step for the simulation was 2 fs. The simulations were run under NPT conditions, using Berendsen's coupling algorithm for keeping the temperature and the pressure constant (P = 1 bar; τ_P_ = 0.5 ps; T = 300 K; τ_T_ = 0.1 ps) [42]. VDW and short range electrostatic forces were treated using a cutoff of 1.2 nm. All simulations were carried out with periodic boundary conditions, using the particle mesh Ewald (PME) method [43] for long range electrostatic forces. During the MD runs, the LINCS algorithm [44] was used in order to constrain the lengths of all bonds; the water molecules were constrained using the SETTLE algorithm [45]. Coordinates were saved every 1 ps. Protein images in Figure 6 were generated using VMD [46].

The diffusion coefficients were calculated from the mean square displacement (MSD) curves between specific time points selected to give the most reliable results. For Ca^2+^ at specific shells from the protein, the MSD statistics used for calculation of the diffusion constant was such that it contained at least 8 different simulations which contributed to the MSD average and the MSD graph was near linear. The diffusion coefficient errors stated in the manuscript are estimates based on multiple calculations using different sets of simulations.

Residence times were calculated by fitting single and double exponential functions to the residence time decay curves. The residence time decay curves denote the number of times a Ca^2+^ ion was found at the desired distance from the protein up to a given time span marked by the he abscissa. In these calculations an event where the Ca^2+^ ion changed its distance from the protein's surface for a period of 2 ps or less was not considered as termination of its residence period. All Apo-CaB simulations were used to generate these curves. For the exponential fitting procedure, only sections of the graph with a count higher than 10 were considered.

Electrostatic potential calculation were performed using the APBS software 1.2.1 [47], with a grid spacing of 1.096×1.096×1.096 Å. The calculations were carried out for a solution having an ionic strength of 150 mM. The dielectric constant of the protein was set as 2 and solvent dielectric of 78.54.

## Supporting Information

Table S1 Total binding times, primary hit count and secondary hit count of Ca^2+^ ion to carbonyl oxygen atoms, calculated from ten 100 ns WT and ten 100 ns E60D simulations.
(0.06 MB DOC)

Table S2 Total binding times, primary hit count and secondary hit count of Ca^2+^ ion to side chain oxygens of charged and polar residues (carboxylate oxygens treated as one), calculated from ten 100 ns WT and ten 100 ns E60D simulations.
(0.07 MB DOC)

## References

[1] Berridge MJ, Lipp P, Bootman MD (2000). The versatility and universality of calcium
signalling.. Nat Rev Mol Cell Biol.

[2] Falke JJ, Drake SK, Hazard AL, Peersen OB (1994). Molecular tuning of ion binding to calcium signaling
proteins.. Q Rev Biophys.

[3] Kretsinger RH, Nockolds CE (1973). Carp muscle calcium-binding protein. II. Structure determination
and general description.. J Biol Chem.

[4] Eigen M (1965). Fast elementary steps in chemical reaction
mechanisms.. Pure & Appl Chem.

[5] Martin SR, Linse S, Johansson C, Bayley PM, Forsen S (1990). Protein surface charges and Ca2+ binding to individual sites
in calbindin D9k: stopped-flow studies.. Biochemistry.

[6] Linse S, Brodin P, Johansson C, Thulin E, Grundstrom T (1988). The role of protein surface charges in ion
binding.. Nature.

[7] Marcus Y (1985).

[8] Vogtle F, Weber E, Patai S (1980). Crown ethers - complexes and selectivity.. The Chemistry of Functional Groups, Suppl E, Part I.

[9] Cox BG, Schneider H (1992). Coordination and transport properties of macrocyclic compounds in
solution.

[10] Kuboniwa H, Tjandra N, Grzesiek S, Ren H, Klee CB (1995). Solution structure of calcium-free calmodulin.. Nat Struct Biol.

[11] Zhang M, Tanaka T, Ikura M (1995). Calcium-induced conformational transition revealed by the
solution structure of apo calmodulin.. Nat Struct Biol.

[12] Ishida H, Nakashima K, Kumaki Y, Nakata M, Hikichi K (2002). The solution structure of apocalmodulin from Saccharomyces
cerevisiae implies a mechanism for its unique Ca2+ binding
property.. Biochemistry.

[13] Seaton BA, Head JF, Engelman DM, Richards FM (1985). Calcium-induced increase in the radius of gyration and maximum
dimension of calmodulin measured by small-angle X-ray
scattering.. Biochemistry.

[14] Komeiji Y, Ueno Y, Uebayasi M (2002). Molecular dynamics simulations revealed Ca(2+)-dependent
conformational change of Calmodulin.. FEBS Lett.

[15] Project E, Friedman R, Nachliel E, Gutman M (2006). A molecular dynamics study of the effect of Ca2+ removal on
calmodulin structure.. Biophysical Journal.

[16] Project E, Friedman R, Nachliel E, Gutman M (2006). A molecular dynamics study of the effect of Ca2+ removal on
calmodulin structure.. Biophys J.

[17] Friedman R, Nachliel E, Gutman M (2005). Molecular dynamics of a protein surface: Ion-residues
interactions.. Biophysical Journal.

[18] Patargias GN, Harris SA, Harding JH A demonstration of the inhomogeneity of the local dielectric
response of proteins by molecular dynamics simulations.. The Journal of Chemical Physics.

[19] Szebenyi DM, Moffat K (1986). The refined structure of vitamin D-dependent calcium-binding
protein from bovine intestine. Molecular details, ion binding, and
implications for the structure of other calcium-binding
proteins.. J Biol Chem.

[20] da Silva AC, Reinach FC (1991). Calcium binding induces conformational changes in muscle
regulatory proteins.. Trends Biochem Sci.

[21] Forsen S, Kordel J, Grundstrom T, Chazin WJ (1993). The molecular anatomy of a calcium-binding
Protein.. Acc Chem Res.

[22] Linse S, Bylsma NR, Drakenberg T, Sellers P, Forsen S (1994). A Calbindin D-9k Mutant with Reduced Calcium Affinity and
Enhanced Cooperativity - Metal-Ion Binding, Stability, and Structural
Studies.. Biochemistry.

[23] Klapper I, Hagstrom R, Fine R, Sharp K, Honig B (1986). Focusing of electric fields in the active site of Cu-Zn
superoxide dismutase: effects of ionic strength and amino-acid
modification.. Proteins.

[24] Venable RM, Pastor RW (1988). Frictional Models for Stochastic Simulations of
Proteins.. Biopolymers.

[25] Kuntz ID, Kauzmann W, Anfinsen CB, Edsall JT, Richards FM (1974). Hydration of Proteins and Polypeptides.. Advances in Protein Chemistry.

[26] David RL (2010). CRC Handbook of Chemistry and Physics 90th
edition..

[27] Robinson RA, Stokes RH (1959). Electrolyte Solutions. 2nd ed.

[28] Szebenyi DME, Moffat K (1986). The Refined Structure of Vitamin-D-Dependent Calcium-Binding
Protein from Bovine Intestine - Molecular Details, Ion Binding, and
Implications for the Structure of Other Calcium-Binding
Proteins.. Journal of Biological Chemistry.

[29] Project E, Nachliel E, Gutman M (2010). Force Field-Dependant Structural Divergence Revealed During Long
Time Simulations of Calbindin d9k.. Journal of Computational Chemistry.

[30] Forsen S, Kordel J, Grundstrom T, Chazin WJ (1993). The Molecular Anatomy of a Calcium-Binding
Protein.. Accounts of Chemical Research.

[31] Kordel J, Pearlman DA, Chazin WJ (1997). Protein solution structure calculations in solution: Solvated
molecular dynamics refinement of calbindin D-9k.. Journal of Biomolecular Nmr.

[32] Paquin R, Ferrage F, Mulder FAA, Akke M, Bodenhausen G (2008). Multiple-Timescale Dynamics of Side-Chain Carboxyl and Carbonyl
Groups in Proteins by C-13 Nuclear Spin Relaxation.. Journal of the American Chemical Society.

[33] Cornell WD, Cieplak P, Bayly CI, Gould IR, Merz KM (1995). A 2Nd Generation Force-Field for the Simulation of Proteins,
Nucleic-Acids, and Organic-Molecules.. Journal of the American Chemical Society.

[34] Sorin EJ, Pande VS (2005). Exploring the helix-coil transition via all-atom equilibrium
ensemble simulations.. Biophysical Journal.

[35] Project E, Nachliel E, Gutman M (2008). Parameterization of Ca+2-protein interactions for molecular
dynamics simulations.. J Comput Chem.

[36] Jorgensen WL, Chandrasekhar J, Madura JD, Impey RW, Klein ML (1983). Comparison of Simple Potential Functions for Simulating Liquid
Water.. Journal of Chemical Physics.

[37] Berendsen HJC, van der Spoel D, van Drunen R (1995). GROMACS: A message-passing parallel molecular dynamics
implementation.. Comp Phys Comm.

[38] Lindahl E, Hess B, Van der Spoel E (2001). GROMACS 3.0:a package for molecular simulation and trajectory
analysis.. J Mol Model.

[39] Van der Spoel D, Lindahl E, Hess B, Groenhof G, Mark AE (2005). GROMACS: Fast, flexible, and free.. Journal of Computational Chemistry.

[40] Hess B, Kutzner C, van der Spoel D, Lindahl E (2008). GROMACS 4: Algorithms for highly efficient, load-balanced, and
scalable molecular simulation.. Journal of Chemical Theory and Computation.

[41] Berman HM, Westbrook J, Feng Z, Gilliland G, Bhat TN (2000). The Protein Data Bank.. Nucleic Acids Res.

[42] Berendsen HJC, Postma JPM, DiNola A, Haak JR (1984). Molecular dynamics with coupling to an external
bath.. J Chem Phys.

[43] Essman U, Perela L, Berkowitz ML, Darden T, Lee H (1995). A smooth particle mesh Ewald method.. J Chem Phys.

[44] Hess B, Bekker H, Berendsen HJC, JGEM. F (1997). LINCS: A linear constraint solver for molecular
simulations.. J Comp Chem.

[45] Miyamoto S, Kollman PA (1992). SETTLE: An Analytical Version of the SHAKE and RATTLE Algorithms
for Rigid water models.. J Comp Chem.

[46] Humphrey W, Dalke A, Schulten K (1996). VMD: Visual molecular dynamics.. Journal of Molecular Graphics.

[47] Baker NA, Sept D, Joseph S, Holst MJ, McCammon JA (2001). Electrostatics of nanosystems: application to microtubules and
the ribosome.. Proc Natl Acad Sci U S A.

