# Supplementary material for: The Dynamics of Ca2+ Ions within the Solvation Shell of Calbindin D9k
Source: PLoS One. 2011 Feb 22;6(2):e14718. doi: 10.1371/journal.pone.0014718 (PMC3043054; doi:10.1371/journal.pone.0014718)
Supplement: Table S1 — Total binding times, primary hit count and secondary hit count of Ca2+ ion to carbonyl oxygen atoms, calculated from ten 100 ns WT and ten 100 ns E60D simulations. (0.06 MB DOC) [file pone.0014718.s001.doc]

| **Table S1. Total binding times, primary hit count and secondary hit count of Ca^2+^ ion to carbonyl oxygen atoms, calculated from ten 100 ns WT and ten 100 ns E60D simulations.** | | | | | | |
| --- | --- | --- | --- | --- | --- | --- |
| **Residue** | **WT** | | | **E60D** | | |
|  | **Binding time (ps)** | **Primary encounters** | **Secondary encounters** | **Binding time (ps)** | **Primary encounters** | **Secondary encounters** |
| A15 | 0 | 0 | 0 | 1080 | 5 | 1 |
| K16 | 2966 | 4 | 2 | 3296 | 5 | 2 |
| E17 | 18903 | 16 | 3 | 18593 | 16 | 5 |
| G18 | 1434 | 5 | 1 | 2878 | 4 | 0 |
| N21 | 0 | 0 | 0 | 13 | 0 | 1 |
| L23 | 55 | 19 | 1 | 0 | 0 | 0 |
| K41 | 657 | 0 | 3 | 722 | 3 | 1 |
| G42 | 841 | 9 | 2 | 1935 | 3 | 1 |
| P43 | 89505 | 16 | 11 | 31363 | 8 | 2 |
| D47 | 2909 | 0 | 7 | 3127 | 1 | 5 |
| E51 | 19566 | 4 | 3 | 76215 | 3 | 0 |
| E52 | 1 | 0 | 1 | 428 | 3 | 1 |
| L53 | 0 | 0 | 0 | 2 | 0 | 1 |
| D54 | 68956 | 1 | 8 | 78190 | 3 | 3 |
| K55 | 1427 | 7 | 3 | 8392 | 16 | 2 |
| N56 | 22530 | 17 | 7 | 4203 | 2 | 7 |
| G57 | 73675 | 28 | 11 | 7737 | 11 | 2 |
| D58 | 146435 | 17 | 23 | 230493 | 27 | 27 |
| G59 | 20194 | 9 | 10 | 486 | 2 | 7 |
| E60 | 25648 | 2 | 3 | 114283 | 9 | 14 |
| E64 | 2 | 0 | 1 | 0 | 0 | 0 |
| K72 | 0 | 0 | 0 | 56253 | 15 | 2 |
| I73 | 22122 | 6 | 3 | 7630 | 15 | 2 |
| S74 | 2531 | 16 | 2 | 11640 | 33 | 6 |
