# Supplementary material for: The Dynamics of Ca2+ Ions within the Solvation Shell of Calbindin D9k
Source: PLoS One. 2011 Feb 22;6(2):e14718. doi: 10.1371/journal.pone.0014718 (PMC3043054; doi:10.1371/journal.pone.0014718)
Supplement: Table S2 — Total binding times, primary hit count and secondary hit count of Ca2+ ion to side chain oxygens of charged and polar residues (carboxylate oxygens treated as one), calculated from ten 100 ns WT and ten 100 ns E60D simulations. (0.07 MB DOC) [file pone.0014718.s002.doc]

| **Table S2. Total binding times, primary hit count and secondary hit count of Ca^2+^ ion to side chain oxygens of charged and polar residues (carboxylate oxygens treated as one), calculated from ten 100 ns WT and ten 100 ns E60D simulations.** | | | | | | |
| --- | --- | --- | --- | --- | --- | --- |
| **Residue** | **WT** | | | **E60D** | | |
|  | **Binding time (ps)** | **Primary encounters** | **Secondary encounters** | **Binding time (ps)** | **Primary encounters** | **Secondary encounters** |
| E4 | 4681 | 5 | 0 | 3282 | 4 | 0 |
| E5 | 1 | 1 | 0 | 0 | 0 | 0 |
| E11 | 3769 | 6 | 1 | 1331 | 1 | 1 |
| Y13 | 0 | 0 | 0 | 1 | 0 | 1 |
| E17 | 128245 | 35 | 12 | 252322 | 41 | 7 |
| D19 | 15339 | 10 | 7 | 1520 | 8 | 2 |
| N21 | 4491 | 4 | 2 | 45288 | 6 | 3 |
| Q22 | 67512 | 52 | 16 | 176169 | 37 | 18 |
| S24 | 1463 | 3 | 8 | 21335 | 15 | 11 |
| E26 | 18961 | 6 | 5 | 18842 | 8 | 6 |
| E27 | 24807 | 4 | 5 | 29762 | 6 | 5 |
| S44 | 3254 | 4 | 10 | 1469 | 2 | 2 |
| T45 | 1874 | 0 | 2 | 1 | 0 | 1 |
| D47 | 62368 | 37 | 11 | 40003 | 24 | 5 |
| E48 | 176802 | 55 | 7 | 92732 | 10 | 7 |
| E51 | 146257 | 58 | 14 | 116178 | 39 | 9 |
| E52 | 12893 | 15 | 2 | 3438 | 6 | 0 |
| D54 | 71503 | 16 | 22 | 18303 | 16 | 4 |
| A56 | 26696 | 11 | 9 | 81417 | 15 | 8 |
| D58 | 82698 | 41 | 24 | 298564 | 61 | 22 |
| E60 | 244019 | 69 | 35 | 442820 | 156 | 29 |
| S62 | 1033 | 14 | 16 | 3118 | 175 | 14 |
| E64 | 31632 | 26 | 10 | 17635 | 28 | 2 |
| E65 | 157857 | 26 | 12 | 21236 | 24 | 7 |
| Q67 | 0 | 0 | 0 | 1 | 0 | 1 |
| Q75 | 80 | 1 | 0 | 1238 | 2 | 1 |
